# The negative effect of *Akkermansia muciniphila*-mediated post-antibiotic reconstitution of the gut microbiota on the development of colitis-associated colorectal cancer in mice

**DOI:** 10.3389/fmicb.2022.932047

**Published:** 2022-10-14

**Authors:** Kaicen Wang, Wenrui Wu, Qing Wang, Liya Yang, Xiaoyuan Bian, Xianwan Jiang, Longxian Lv, Ren Yan, Jiafeng Xia, Shengyi Han, Lanjuan Li

**Affiliations:** ^1^State Key Laboratory for Diagnosis and Treatment of Infectious Diseases, National Clinical Research Center for Infectious Diseases, Collaborative Innovation Center for Diagnosis and Treatment of Infectious Diseases, The First Affiliated Hospital, College of Medicine, Zhejiang University, Hangzhou, China; ^2^Jinan Microecological Biomedicine Shandong Laboratory, Jinan, China

**Keywords:** *Akkermansia muciniphila*, colitis-associated CRC, microbiome, gut microbial reconstruction, gut barrier, inflammation, bile acids metabolism, short-chain fatty acids

## Abstract

The bidirectional relationship between colorectal cancer (CRC) and the gut microbiome has been well-documented. Here, we investigated the impact of *Akkermansia muciniphila*-mediated post-antibiotic gut microbial reconstitution on the development of colitis-associated CRC (CAC). The results showed that post-antibiotic replenishment of *A. muciniphila* worsened the tumorigenesis of CAC as indicated by increased number of large (>2 mm in diameter) tumors and both average and total tumor diameters. Measures of intestinal barrier function showed that post-antibiotic *A. muciniphila* gavage damaged the intestinal barrier as reflected by lower transcriptional levels of *Tjp1, Ocln, Cdh1*, and *MUC2*. Impaired gut barrier was followed by lipopolysaccharides (LPS) translocation as indicated by higher level of serum LPS-binding protein (LBP). The increased colonic mRNA levels of *Il1b, Il6*, and *Tnfa* and serum levels of IL-1β, IL-6, and TNF-α indicated that post-antibiotic *A. muciniphila* replenishment resulted in overactivated inflammatory environment in CAC. The analysis of the evolution of the microbial community during the progression of CAC showed that post-antibiotic supplementation of *A. muciniphila* led to a distinct microbial configuration when compared with other treatments characterized by enriched Firmicutes, Lachnospiraceae, and Ruminococcaceae, and depleted Bacteroidetes, which was accompanied by higher Firmicutes/Bacteroidetes (F/B) ratio. Furthermore, post-antibiotic *A. muciniphila* administration changed the bile acid (BA) metabolic profile as indicated by decreased concentrations of secondary BA (SBA), ω–murocholic acid (ωMCA), and murocholic acid (muroCA). In addition, the *A. muciniphila* supplementation after antibiotic pretreatment also impacted the metabolism of short-chain fatty acids (SCFAs) as evidenced by increased concentrations of acetic acid, propionic acid, butyric acid, and valeric acid. Our study surprisingly observed that *A. muciniphila*-mediated post-antibiotic reconstitution of the gut microbiota aggravated the CAC in mice. It might exert its effect by damaging the gut barrier, exacerbating inflammatory responses, disrupting the post-antibiotic recovery of the microbial community, and further influencing the metabolism of BA and SCFAs. These findings indicated that maintaining the homeostasis of intestinal microorganisms is more crucial to health than replenishing a single beneficial microbe, and probiotics should be used with caution after antibiotic treatment.

## Introduction

Colorectal cancer (CRC) is the third most commonly diagnosed cancer and the second leading cause of cancer-related deaths worldwide (Sung et al., 2021). CRC has long been considered to be a type of chronic inflammation-associated tumor, and intestinal inflammation begins at the very beginning of tumor development. Prolonged inflammation in the intestine, as what occurs in inflammatory bowel disease (IBD), poses a greater risk for colonic tumorigenesis, which is termed colitis-associated CRC (CAC) (Nadeem et al., 2020; Wang et al., 2021). The human microbiome contains approximately 10^14^ bacteria, which is almost equal to the number of cells in an individual (Eckburg et al., 2005). Most host microbes reside in the gastrointestinal (GI) tract, especially in the colon (Ley et al., 2006). Gut microbial dysbiosis, namely, changes in the composition and distribution of intestinal microbes and their metabolic activities was closely related to increased risks of IBD and CRC (Chen et al., 2017; Larabi et al., 2020).

Antibiotics are prescribed for treatment of bacterial infections (Van Boeckel et al., 2014). However, extensive antibiotic use is inevitably accompanied by disruption in the composition of the commensal microbial community and subsequently causes microbial dysbiosis (Bartlett, 2002). Once antibiotics are stopped, the human microbiome undergoes a dynamic rebuilding process, which is often slow and incomplete (Jernberg et al., 2007; Dethlefsen et al., 2008; Dethlefsen and Relman, 2011). In some cases, it takes even years for the microbiome to revert to its naïve configuration (Lankelma et al., 2017). Some studies on humans and rodent models suggest an association between antibiotic exposure, especially during the early stages of life, and susceptibility to IBD (Kronman et al., 2012; Virta et al., 2012). Nevertheless, few studies have focused on the impact of post-antibiotic reconstitution of the gut microbiome on the onset and development of diseases.

Probiotics are widely applied for prevention of antibiotic-associated dysbiosis and its related adverse effects in some human (Hempel et al., 2012; Allen et al., 2013; Olek et al., 2017) and rodent studies (Ekmekciu et al., 2017). *Akkermansia muciniphila*, a novel mucin-degrading bacterium, was first isolated from healthy human feces in 2004 by Derrien et al. (2004). It is the first and only representative member of the Verrucomicrobia found in the human intestinal tract (Miller and Hoskins, 1981; Derrien et al., 2010). As an abundant resident in the GI tract, *A. muciniphila* accounts for 1–3% of the whole fecal bacterial community (Derrien et al., 2008). Recently, this bacterium has been considered as a promising next-generation probiotic. The abundance of *A. muciniphila* was shown to be significantly decreased in patients with UC and mice with colitis or CAC (Zhang et al., 2017; Wang et al., 2020). Our previous study has also shown that supplementation with *A. muciniphila* improved the colonic inflammation index and colonic histological score in acute colitis (Bian et al., 2019). These results established the potential benefit of *A. muciniphila* in treatment of colitis and CAC.

The aim of our study was to investigate the impact of *A. muciniphila*-mediated post antibiotic reconstitution of the gut microbiome on the initiation and development of azoxymethane (AOM)/dextran sodium sulfate (DSS)-induced CAC in a mouse model.

## Materials and methods

### Animal experiments

**FIGURE 1:**
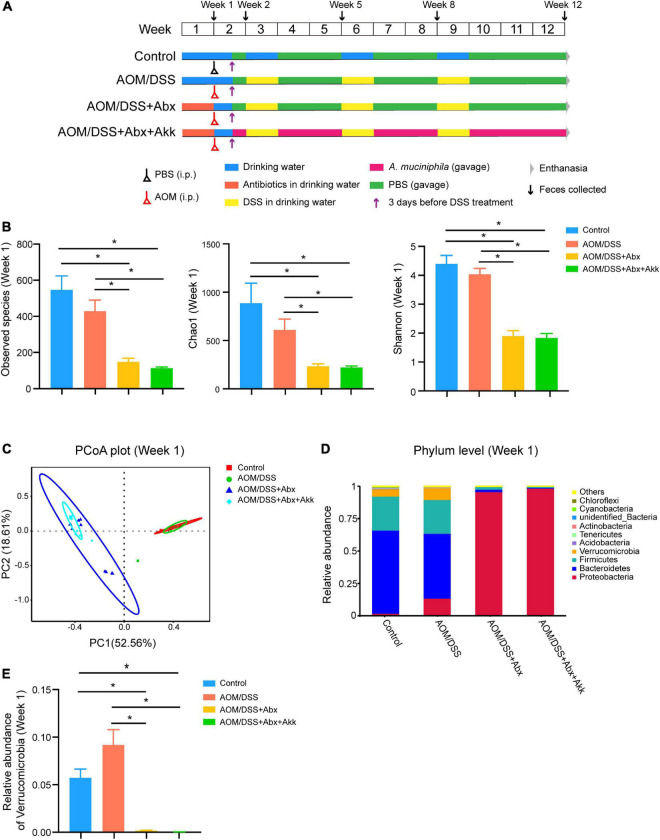
Antibiotic treatment significantly disrupted the intestinal microbiome. **(A)** Experimental schema. Control group, normal control mice; AOM/DSS group, AOM/DSS-induced CAC mice; AOM/DSS + Abx group, AOM/DSS-induced CAC mice pretreated with the antibiotic cocktail; AOM/DSS + Abx + Akk group, AOM/DSS-induced CAC mice pretreated with the antibiotic cocktail and further administrated with *Akkermansia muciniphila* during tumorigenesis. **(B)** α-Diversity indexes (observed species, Chao1, Shannon) of the gut microbiota among the four groups (week 1). **(C)** PCoA plot based on Bray-Curtis distance of the four groups (week 1). **(D)** Relative abundance of the top ten most abundant taxa at the phylum level of the four groups (week 1). **(E)** Relative abundance of Verrucomicrobia among the four groups (week 1). Data are shown as means ± SEM, *P < 0.05.

Male C57BL/6 mice 6–8 weeks of age were housed with free access to food and water. The mice were divided into four groups (8–12 mice per group) after 1 week of acclimation: the normal control group (Control), the AOM/DSS-induced CAC group (AOM/DSS), the CAC group pretreated with an antibiotic cocktail (AOM/DSS + Abx), and the CAC group pretreated with a cocktail of antibiotics and further supplemented with *A. muciniphila* during tumorigenesis (AOM/DSS + Abx + Akk). In the AOM/DSS + Abx and AOM/DSS + Abx + Akk groups, mice were administrated a combination of four antibiotics (ampicillin 200 mg/L, vancomycin 100 mg/L, neomycin 200 mg/L, and metronidazole, 200 mg/L) through their drinking water for seven consecutive days before AOM injection. To establish the CAC model, the mice in the AOM/DSS, AOM/DSS + Abx, and AOM/DSS + Abx + Akk groups were challenged with a single dose of AOM intraperitoneal injection (12.5 mg/kg, Sigma-Aldrich, St. Louis, MO, United States) on the 8th day. Then, 1 week after the AOM injection, three 7-day cycles of 2% DSS (36,000–50,000 Da, MP Biomedicals, CA, United States) in drinking water were performed with 14-day recovery periods between consecutive DSS cycles. The mice were sacrificed 3 weeks after the last DSS treatment. The mice in the AOM/DSS + Abx + Akk group were orally administrated with 200 μl of viable *A. muciniphila* (3 × 10^∧9^ CFU) every day from 3 days before the DSS treatment to sacrifice but skipped the DSS treatment periods. Body weight was monitored during the experimental period, and feces were collected on weeks 1, 2, 5, and 8 and before sacrifice (week 12) (refer to Figure 1A for the experimental schema).

### Culture of *Akkermansia muciniphila*

*Akkermansia muciniphila* Muc*^T^* (ATTC BAA835) was cultivated in a basal mucin-based brain heart infusion (BHI) medium under strict anaerobic conditions (37°C, 48 h). After that, bacterial enrichments were centrifuged at 4°C (8,000 rpm, 15 min), washed with sterile PBS, and resuspended to a final concentration of 1.5 × 10^∧10^ colony-forming units (CFUs)/ml by determining the optical density (OD) value at 630 nm. Then, 200 μl of a bacterial solution (3 × 10^∧9^ CFU) was prepared for oral administration to each mouse.

### Histology and immunohistochemistry staining

Colon sections (tumor segments or normal tissues) were collected carefully and immediately fixed in 10% formalin. After paraffin embedding, the tissues were prepared at 5 μm thickness for hematoxylin and eosin (H&E) staining to examine the colon pathology. For immunohistochemical (IHC) staining, colonic specimens were stained with an anti-Ki-67 antibody to evaluate the proliferation of colonic cells. Images were visualized with P250 FLASH (3D HISTECH, Budapest, Hungary), and the area of Ki-67 antigen-positive cells was calculated using QUANT CENTER.

### Serum cytokine detection

A 23-plex assay kit (Bio-Plex Pro Mouse Cytokine 23-Plex Panel, Bio-Rad, Hercules, CA, United States) was used to detect the concentration of serum cytokines following the manufacturer’s instructions. The data were further analyzed using a MAGPIX system (Luminex Corporation, Austin, TX, United States) and the Bio-Plex Manager 6.1 software (Bio-Rad, Hercules, CA, United States).

### RNA extraction and real-time PCR analysis

RNeasy Mini Kit (Qiagen, Hilden, Germany) was used to extract the total RNA from the colon samples according to the product manual. The extracted total RNA was immediately reverse-transcribed to cDNA, and the cDNA was then stored at −80°C for further use. mRNA relative levels were measured in duplicate with TB Green Premix Ex Taq Reagent (TAKARA, Kusatsu, Japan) using an Applied Biosystems VIIA7 Real-time PCR system (Applied Biosystems, Waltham, MA, United States) (refer to Supplementary Table 1 for primer information). The relative mRNA expression levels of targeted genes was normalized by GAPDH expression.

### Serum lipopolysaccharides-binding protein assessment

Serum lipopolysaccharides (LPS)-binding protein (LBP) concentration was quantified using mouse LBP ELISA Kit (ab269542, Abcam, Cambridge, UK) according to the manufacturer’s instructions.

### 16S rRNA gene sequencing

The total bacterial genome DNA of the feces was extracted with DNeasyPowerSoil Kit (QIAGEN, Hilden, Germany) following the manufacture’s protocol. The V3–V4 region of the 16S rRNA gene was amplified using universal bacterial primers with a sample-specific barcode (341F: 5′-CCTAYGGGRBGCASCAG-3′ and 806R: 5′-GGACTACNNGGGTATCTAAT-3′). Sequencing libraries were constructed, qualified, and sequenced on a NovaSeq 6000 (PE 250) platform. Raw sequencing reads with exact match to the barcodes were assigned to respective samples and identified as valid sequences. Paired-end reads were merged using FLASH (V1.2.7) and then filtered under specific filtering conditions to obtain high-quality clean sequences according to the QIIME (V1.7.0) quality control process. After chimera removal, effective tags were clustered into OTUs at 97% sequence identity using the Usearch software (Usearch v10). For each representative sequence, the GreenGene Database was used based on an RDP classifier (Version 2.2) to annotate the taxonomic information of each representative sequence within each OTU. Then, a statistical analysis of microbial diversity and differential enrichment was conducted.

### Fecal bile acid profiling

Fecal bile acid (BA) was extracted through a two-step process. Twenty-five mg of precooled mill beads and 200 μl acetonitrile/methanol (v/v = 8:2, containing 10 μl internal standards GCA-d4, TCA-d4, TCDCA-d9, UDCA-d4, CA-d4, GCDCA-d4, GDCA-d4, DCA-d4, LCA-d4, and b-CA-d5, each for 150 nM) were added to tubes containing 10 mg feces, homogenized, and then centrifuged at 4°C (13,500 rpm, 20 min). Ten μl of supernatant was diluted with 90 μl acetonitrile/methanol (v/v = 8:2) mixed solvent/ultrapure water (v/v = 1:1); after vortexing, the diluent was centrifuged at 4°C (13,500 rpm, 20 min). Five μl of the supernatant was transferred to a sampling vial and analyzed for BA quantification by ultraperformance liquid chromatography coupled to a tandem mass spectrometry (UPLC-MS/MS, ACQUITY UPLC-Xevo TQ-S; Waters Corp., Milford, MA, United States). The calibration standard solution at 7 different concentration levels containing 23 standards was tested. The raw data were subjected to processing, including peak annotation and quantitation, on QuanMET (v1.0, Metabo-Profile, Shanghai, China).

### Fecal short-chain fatty acids analysis

Short-chain fatty acids (SCFAs) (including acetic acid, propionic acid, isobutyric acid, butyric acid, 2-methylbutyric acid, and valeric acid) in the fecal samples were assessed according to the protocol we have previously established (1). Briefly, 500 μl internal standard (hexanoic acid-d3 at 10 μg/ml) was added to the tubes containing 20 mg feces, homogenized, and then centrifuged at 4°C (15,000 rpm, 5 min). The supernatant was transferred into a new tube, and 1/10 volume of 5% concentrated sulfuric acid and 1 volume of ethyl acetate were added to the tube and vortexed. The mixed solution was centrifuged at 4°C (15,000 rpm, 5 min) and then incubated at 4°C away from light for 30 min. One hundred twenty microliters of the supernatant was transferred to a chromatographic vial with a 150-μl internal tube, and metabolites were analyzed by gas chromatography mass spectrometry (GC/MS; Agilent Technologies, Santa Clara, CA, United States). The calibration standard solution at 6 different concentration levels containing six standards was tested.

### Statistical analysis

GraphPad Prism 8 and R language (R 3.6.3) were used for statistical analyses. Data were presented as means ± SEM. One-way ANOVA with Tukey’s *post hoc* test or Mann-Whitney *U* test was conducted for the analyses. *P* value < 0.05 was considered significant.

## Results

### Antibiotics treatment resulted in gut microbial depletion

Researchers have often used antibiotics to deplete or disrupt the gut microbial community to better understand its role in pathological states (Rakoff-Nahoum et al., 2004; Shen et al., 2015; Sampson et al., 2016). In our study, we administrated a cocktail containing four broad-spectrum antibiotics (vancomycin, neomycin, ampicillin, and metronidazole) to mice through drinking water to deplete the intestinal microbiota.

Seven days of antibiotic cocktail treatment sharply decreased the bacterial load as reflected by the reduced number of observed species in the AOM/DSS + Abx and AOM/DSS + Abx + Akk groups in comparison with the Control and AOM/DSS groups (Figure 1B). In addition, the antibiotic treatment also significantly reduced the species richness and diversity as indicated by the dramatically lower levels of the Chao1 and Shannon indexes, respectively, in the AOM/DSS + Abx and AOM/DSS + Abx + Akk groups compared to the Control and AOM/DSS groups (Figure 1B). The principal coordinate analysis (PCoA) based on Bray-Curtis distance demonstrated that the first two principal components explained >70% of the variation, clearly separating the antibiotic-treated groups (AOM/DSS + Abx and AOM/DSS + Abx + Akk) from the non-antibiotic treated groups (Control and AOM/DSS) (Figure 1C). The results of Adonis further supported the separation (Supplementary Table 2). The top ten abundant taxa at the phylum level presented in Figure 1D depict that the gut microbiota of mice in the Control and AOM/DSS groups are dominated by Firmicutes and Bacteroidetes at the phylum level, which, combined, account for more than 75% of the total bacteria, followed by Proteobacteria (Control: 2%, AOM/DSS: 14%) and Verrucomicrobia (Control: 6%, AOM/DSS: 10%). However, after 1 week of antibiotic treatment, Proteobacteria governed the gut microbial communities of the AOM/DSS + Abx and AOM/DSS + Abx + Akk groups (96 and 99%, respectively), with Verrucomicrobia being too low to be detected (Figures 1D,E).

Overall, the antibiotic cocktail management significantly decreased the bacterial load and dramatically reshaped the composition of the gut microbial community.

### *Akkermansia muciniphila* replenishment after antibiotics treatment aggravated the outcome of colitis-associated colorectal cancer

**FIGURE 2:**
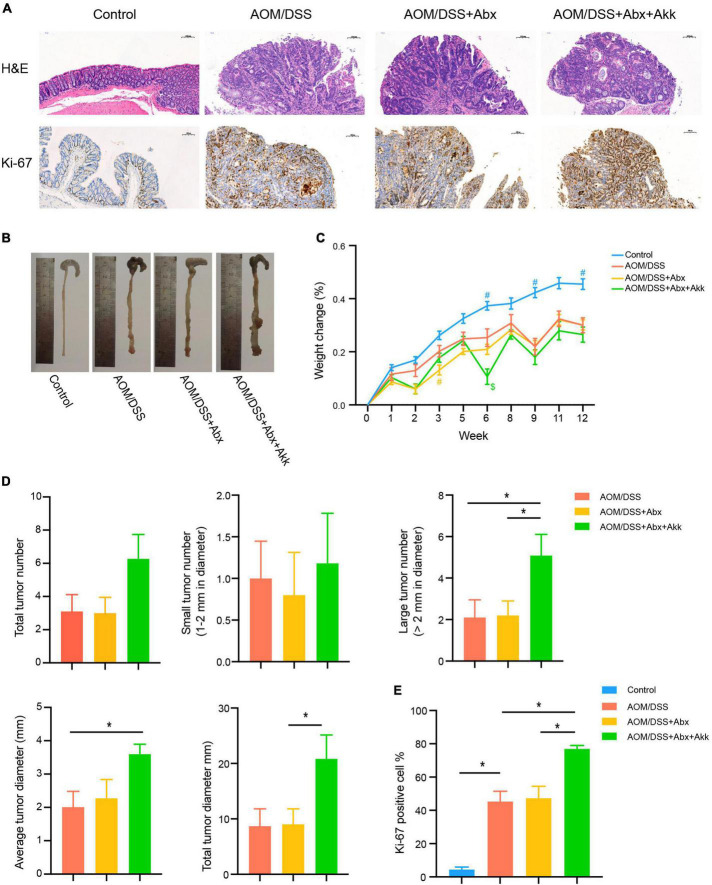
Spontaneous recovery of the gut microbiome had no effect on the outcome of CAC, whereas post-antibiotic replenishment with *Akkermansia muciniphila* worsened the outcome of CAC. **(A)** Representative pathological H&E staining and Ki-67 protein immunohistochemical staining of colon tissues of the four groups. **(B)** Tumor development of the four groups. **(C)** Percentage of weight change from the baseline (week 0) to the end of the experiment (week 12). Data are shown as means ± SEM. The colors of “#” and “$” represent the corresponding groups, blue: Control; yellow: AOM/DSS + Abx; green: AOM/DSS + Abx + Akk. ^#^*P* < 0.05, compared to the AOM/DSS group at the corresponding time point; ^$^*P* < 0.05, compared to the AOM/DSS + Abx group at the corresponding time point. **(D)** Assessment of tumor occurrence in the four groups: total tumor number, small tumor number (1–2 mm in diameter), large tumor number (>2 mm in diameter), average tumor diameter (mm), and total tumor diameter (mm). **(E)** Percentage of Ki-67 protein-positive cells in the four groups. Data are shown as means ± SEM. *P < 0.05.

In the context of disrupted microbiota, CAC was induced in the AOM/DSS + Abx and AOM/DSS + Abx + Akk groups. In addition, the mice in the AOM/DSS + Abx + Akk group was administrated with live *A. muciniphila* during the development of tumorigenesis to investigate the impact of post-antibiotic *A. muciniphila* supplementation on CAC. As shown by the pathological evaluation of H&E-stained images and gross observation, the mice in the AOM/DSS, AOM/DSS + Abx, and AOM/DSS + Abx + Akk groups all developed carcinoma (Figures 2A,B). Body weight was measured during the experimental period, and the percentage of change in body weight from the baseline was calculated. Tumorigenesis was accompanied by significant weight loss in the AOM/DSS, AOM/DSS + Abx, and AOM/DSS + Abx + Akk groups compared to the Control group, whereas similar weight change was observed among the AOM/DSS, AOM/DSS + Abx, and AOM/DSS + Abx + Akk groups (Figure 2C). Additionally, we observed that during the development of tumorigenesis, body weight fluctuated with the presence of DSS in drinking water, which consistently caused weight loss (Figure 2C).

Antibiotic pretreatment for 7 days alone did not influence the future tumor development in the AOM/DSS + Abx group as indicated by the similar total tumor number, small tumor (1–2 mm in diameter) number, large tumor (>2 mm in diameter) number, average and total tumor diameters in the AOM/DSS + Abx group to that in the AOM/DSS group (Figure 2D). Furthermore, the AOM/DSS and AOM/DSS + Abx groups exhibited no difference in proliferation of colon cells as reflected by the similar Ki-67 protein expression between the two groups (Figures 2A,E).

Nevertheless, the post-antibiotic intragastric administration of *A. muciniphila* during the development of CAC unexpectedly exacerbated the tumorigenesis. Although the total tumor and small tumor (1–2 mm in diameter) numbers did not vary between the AOM/DSS + Abx and AOM/DSS + Abx + Akk groups, we surprisingly observed that the number of large tumors (>2 mm in diameter) in the AOM/DSS + Abx + Akk group was more than that in the AOM/DSS + Abx group (Figure 2D). Furthermore, both the average tumor diameter and total tumor diameter per mouse in the AOM/DSS + Abx + Akk group were larger than those in the AOM/DSS + Abx group (Figure 2D). The percentage of Ki-67-protein-positive cells in the colon tissues of the AOM/DSS + Abx + Akk group was significantly increased compared to that of the AOM/DSS + Abx group (Figures 2A,E), indicating that the supplementation with *A. municiphila* after antibiotic pretreatment strongly exacerbated the proliferation of colon cancer cells.

Collectively, the results demonstrated that the very early short-term administration of the antibiotic cocktail alone did not interfere with the outcome of tumorigenesis during the long-term development of CAC, and the post-antibiotic *A. municiphila* supplementation did not slow down the formation of tumors as we initially expected but instead increased the tumor burden.

### Post-antibiotic supplementation with *Akkermansia mucinipihla* worsened the intestinal barrier in the development of colitis-associated colorectal cancer

**FIGURE 3:**
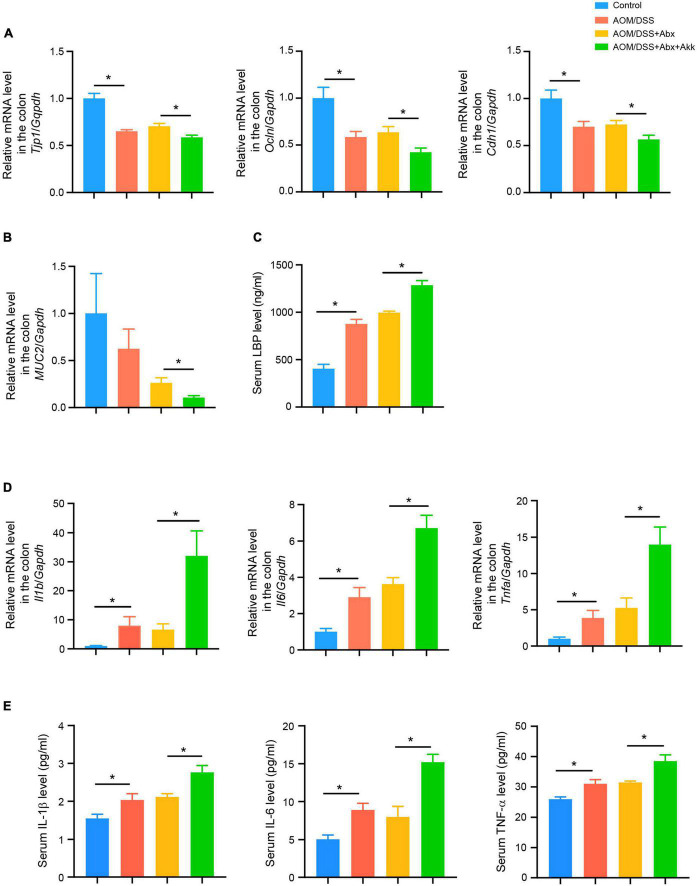
Post-antibiotic supplementation with *Akkermansia muciniphila* worsened the gut barrier and aggravated colonic and systemic inflammation during tumorigenesis. **(A)** Colonic relative mRNA levels of *Tjp1, Ocln*, and *Cdh1* in the four groups. **(B)** Colonic relative mRNA expression level of *MUC2* in the four groups. **(C)** Serum LBP level in the four groups. **(D)** Colonic relative mRNA expression levels of *Il1b, Il6*, and *Tnfa* in the four groups. **(E)** Serum levels of IL-1β, IL-6, and TNF-α in the four groups. Data are shown as means ± SEM. *P < 0.05.

Intestinal barrier disruption is associated with progression of CRC and its severity. Tight junction proteins (occludin, tight junction protein 1) and an adhesion junction protein (E-cadherin) are important components of the mechanical connection between intestinal epithelial cells. These proteins help in maintaining the intestinal epithelial structure as well as protecting the intestinal barrier function. Thus, the relative mRNA expressions of *Tjp1* (the gene encoding tight junction protein 1), *Ocln* (the gene encoding occludin), and *Cdh1* (the gene encoding E-cadherin) in the colon tissues were measured to assess the intestinal barrier function among the four groups. The results showed that the CAC development in the AOM/DSS group was accompanied with destroyed intestinal barrier as indicated by decreases in the transcriptional levels of *Tjp1, Ocln*, and *Cdh1* compared to the Control group (Figure 3A). Besides, the AOM/DSS + Abx group had similar mRNA expression levels of *Tjp1, Ocln*, and *Cdh1* with the AOM/DSS group, indicating that the short period of the antibiotic pretreatment did not further damage the barrier during the progression of tumorigenesis (Figure 3A). However, the AOM/DSS + Abx + Akk group exhibited a more damaged intestinal epithelial barrier, as reflected by the significantly decreased expressions of *Tjp1, Ocln*, and *Cdh1*, than the AOM/DSS + Abx group (Figure 3A). Mucin 2 is the main component of the mucin secreted by intestinal goblet cells, which could stabilize the structure of the mucus barrier and enhance the function of the intestinal mucus barrier. Hence, we further assessed the relative expression of *MUC2* (the gene encoding mucin 2) in the colon tissues and found a decreasing trend of *MUC2* expression in the AOM/DSS group compared to the Control group, albeit with no significant difference (Figure 3B). The same was true for the AOM/DSS and AOM/DSS + Abx groups (Figure 3B). The post-antibiotic supplementation with *A. muciniphila* significantly decreased the expression of *MUC2* in the AOM/DSS + Abx + Akk group compared to the AOM/DSS + Abx group (Figure 3B). These results indicated a more severe gut barrier disruption in the AOM/DSS + Abx + Akk group. The destroyed intestinal barrier could further increase intestinal permeability. Bacteria-derived lipopolysaccharides (LPS) could subsequently translocate from the “leaky gut” to the systemic circulation causing endotoxemia. The LPS-binding protein (LBP) is a well-recognized indicator of serum LPS level (Muta and Takeshige, 2001). We next measured the serum LBP level in the four groups. The AOM/DSS group exhibited higher LBP level than the Control group, indicating increase in intestinal permeability in the development of CAC (Figure 3C). Similar LBP concentrations were observed between the AOM/DSS and AOM/DSS + Abx groups (Figure 3C). The post-antibiotic *A. muciniphila* treatment caused more severe “leaky gut” during the progression of tumorigenesis as evidenced by the significantly increased serum LBP level in the AOM/DSS + Abx + Akk group compared to the AOM/DSS + Abx group (Figure 3C).

These results indicated that the supplementation with *A. muciniphila* after the antibiotic treatment aggravated the impaired intestinal barrier and endotoxemia induced by CAC.

### Post-antibiotic replenishment of *Akkermansia mucinipihla* aggravated colonic and systemic inflammation in the development of colitis-associated colorectal cancer

The LPS in the gut is an important factor in inducing chronic intestinal inflammation, and endotoxemia can also trigger a systemic inflammatory response (Candelli et al., 2021). Thus, the inflammatory environment in both the colon and the systemic circulation was further assessed. The colonic mRNA levels of *Il1b, Il6*, and *Tnfa* in the AOM/DSS group was higher than that in the Control group but similar to that in the AOM/DSS + Abx group (Figure 3D). However, the AOM/DSS + Abx + Akk group showed increased transcriptional levels of *Il1b, Il6*, and *Tnfa* compared to the AOM/DSS + Abx group (Figure 3D). The serum cytokine analysis demonstrated inflammatory activation in the AOM/DSS group with respect to the Control group as indicated by increased concentrations of eotaxin, G-CSF, IL-1β, IL-4, IL-5, IL-6, IL-12(p40), and TNF-α (Figure 3E and Supplementary Table 3). In the AOM/DSS + Abx group, the level of MIP-1a was increased whereas the levels of eotaxin and IL-12(p40) were reduced in contrast to the AOM/DSS group (Supplementary Table 3). The post-antibiotic gavage of *A. muciniphila* significantly elevated the levels of IL-1β, IL-3, IL-6, IL-9, IL-12 (p40), IL-17A, KC, MCP-1, and TNF-α in the AOM/DSS + Abx + Akk group in comparison to the AOM/DSS + Abx group (Figure 3E and Supplementary Table 3).

These results pointed out that the administration of *A. muciniphila* after the antibiotic pretreatment exacerbated the inflammation during tumorigenesis.

### The gut microbial community dynamically changed during colitis-associated tumorigenesis

To investigate dynamic changes in the gut microbial community during the progress of colitis-associated tumorigenesis, 16S rRNA gene sequencing was performed on the feces in different stages of CAC development in the AOM/DSS group.

**FIGURE 4:**
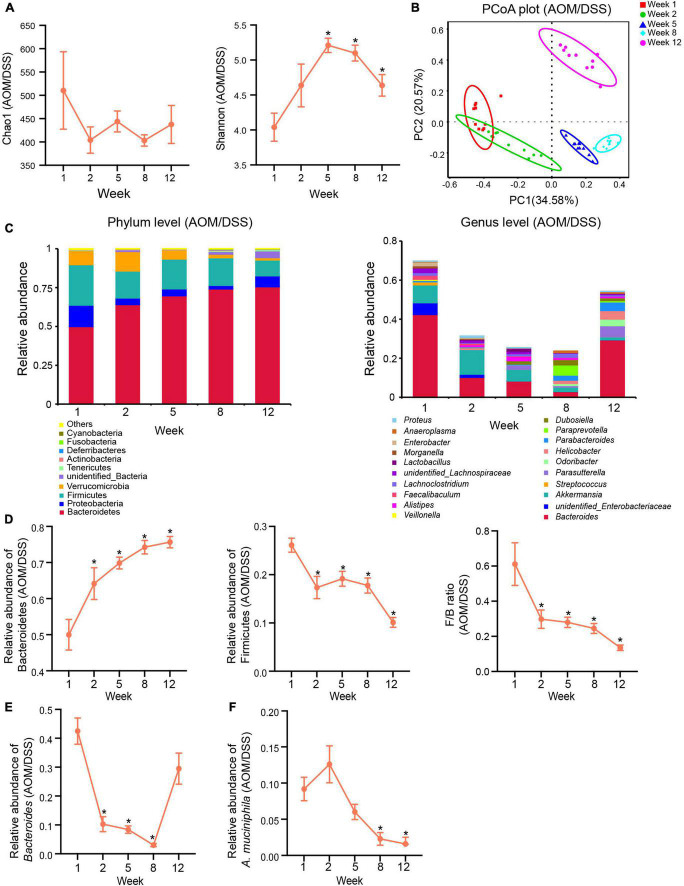
Gut microbiota changed dynamically in the development of CAC in the AOM/DSS group. **(A)** α-Diversity indexes (Chao1 and Shannon) of the gut microbiota in different stages of tumorigenesis. **(B)** PCoA plot based on Bray-Curtis distance showing the dynamical composition changes in the gut microbial community during the development of CAC. **(C)** Relative abundance of the top ten most abundant taxa at the phylum level (left) and top twenty most abundant taxa and genera (right) during the different stages of tumorigenesis. **(D)** Changes in relative abundances of Bacteroidetes and Firmicutes and changes in the ratio of Firmicutes/Bacteroidetes (F/B) in the development of CAC. **(E)** Changes in the relative abundance of *Bacteroides* in the development of CAC. **(F)** Changes in the relative abundance of *A. muciniphila* in the development of CAC. Data are shown as means ± SEM. *P < 0.05. The levels of each index at different time points compared with the corresponding level of week 1. Week 1: baseline, week 2: after AOM injection, week 5: after the first cycle of DSS treatment, week 8: after the second cycle of DSS treatment, and week 12: after the third cycle of DSS treatment and before sacrifice.

The richness of the microbial community, as indicated by the Chao1 index, showed that the number of species displayed a fluctuating downtrend during the tumorigenesis. The diversity of the microbial community, as reflected by the Shannon index, demonstrated that in the development of CAC, microbial diversity was first increased and then decreased (Figure 4A). The PCoA based on Bray-Curtis distance revealed that microbiota structures in different stages of tumor development were distinct from each other in the AOM/DSS group (Figure 4B). The results of Adonis further confirmed the distinguishment (Supplementary Table 4). Figure 4C showed the top most abundant microbial taxa at the phylum and genus levels in the different stages of tumorigenesis in the AOM/DSS group. At the phylum level, we observed consistent increases in the relative abundance of Bacteroidetes and decreases in the relative abundance of Firmicutes accompanied by decreases in the ratio of Firmicutes/Bacteroidetes (F/B) during the progression of CAC (Figure 4D). At the genus level, the relative abundance of *Bacteroides* first decreased sharply before week 8 but increased during the last DSS treatment cycle (Figure 4E). *Akkermansia muciniphila* is the sole representative species of the phylum Verrucomicrobia in the intestine, so the relative abundance of the phylum Verrucomicrobia or the genus *Akkermansia* could be considered the relative abundance of *A. muciniphila*. Interestingly, during the development of tumorigenesis, the relative abundance of *A. muciniphila* decreased in a time-dependent pattern in the AOM/DSS group. After the AOM treatment (week 2), the relative abundance of *A. muciniphila* increased by 37% in the AOM/DSS group compared with its level on week 1 (Figure 4F). Nevertheless, subsequent DSS in the drinking water consistently decreased its abundance (Figure 4F).

### Post-antibiotic administration of *Akkermansia muciniphila* reshaped microbial reconstruction during tumorigenesis

**FIGURE 5:**
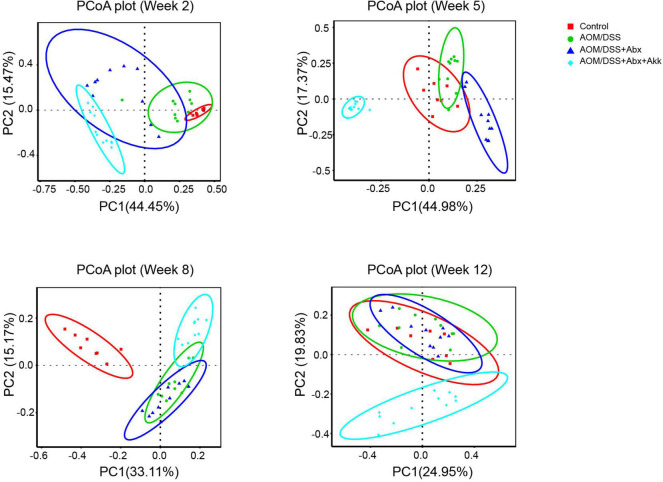
Post-antibiotic administration of *Akkermansia muciniphila* reshaped microbial reconstruction during tumorigenesis. PCoA plot based on Bray-Curtis distance showing the evolution of the gut microbial structure during the development of tumorigenesis in the Control, AOM/DSS, AOM/DSS + Abx, and AOM/DSS + Abx + Akk groups. Week 2: after AOM injection, week 5: after the first cycle of DSS treatment, week 8: after the second cycle of DSS treatment, and week 12: after the third cycle of DSS treatment and before sacrifice.

In the AOM/DSS + Abx group, inflammation-associated tumorigenesis was accompanied by post-antibiotic spontaneous reconstruction of the gut microbiota. The PCoA plot based on Bray-Curtis distance showed that although the microbial compositions of the AOM/DSS + Abx group were different from those of the AOM/DSS group in the early and middle processes, the fecal microbial profile of the AOM/DSS + Abx group could not be discriminated from that of the AOM/DSS group at the end of the experiment (Figure 5), which was further confirmed by the Adonis results (Supplementary Table 5). Further analysis also showed that the microbial communities of the AOM/DSS and AOM/DSS + Abx groups did not differ from each other at different phylogenetic levels.

Nevertheless, the PCoA plot based on Bray-Curtis distance demonstrated that the dynamic reconstruction of the gut microbiota with involvement of *A. muciniphila* obviously shaped a distinct microbial community during the progression of tumorigenesis in the AOM/DSS + Abx + Akk group compared to the Control, AOM/DSS, and AOM/DSS + Abx groups (Figure 5), and it was further supported by the Adonis results (Supplementary Table 5).

**FIGURE 6:**
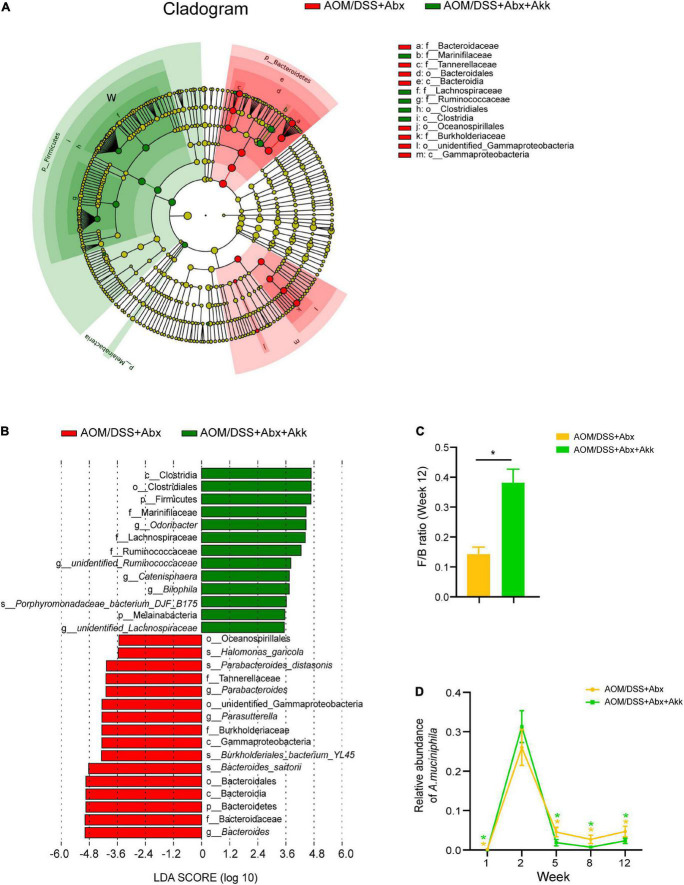
*Akkermansia muciniphila* supplementation after antibiotic treatment shaped a distinct microbial profile at the end of the experiment (week 12). **(A)** LEfSe cladogram representing taxon enrichment in the AOM/DSS + Abx and AOM/DSS + Abx + Akk groups. **(B)** Discriminative microbial biomarkers with an LDA score of >3.5 or <-3.5 in the AOM/DSS + Abx and AOM/DSS + Abx + Akk groups. **(C)** F/B ratio (week 12) in the AOM/DSS + Abx and AOM/DSS + Abx + Akk groups. **(D)** changes in the relative abundances of *A. muciniphila* in the AOM/DSS + Abx and AOM/DSS + Abx + Akk groups. Data are shown as means ± SEM. *P < 0.05. The asterisks on the line graph in panel D represent the results of comparing the *A. muciniphila* abundance of each group at each time point with the corresponding abundance on week 2. Colors of the asterisks represent different groups (yellow: AOM/DSS + Abx and green: AOM/DSS + Abx + Akk).

A linear discriminant analysis (LDA) effect size (LEfSe) analysis at different phylogenetic levels was performed to further explore the characteristic microbes between the AOM/DSS + Abx and AOM/DSS + Abx + Akk groups at the end of the experiment. The AOM/DSS + Abx group enriched the Bacteroidetes at the phylum level; Bacteroidaceae, Burkholderiaceae, and Tannerellaceae at the family level; *Bacteroides, Parasutterella*, and *Parabacteroides* at the genus level; *Bacteroides sartorii, Burkholderiales bacterium_YL45, Parabacteroides distasonis*, and *Halomonas garicola* at the species level (Figures 6A,B), whereas the AOM/DSS + Abx + Akk group enriched Firmicutes and Melainabaceteria at the phylum level; Marinifilaceae, Lachnospiraceae, and Ruminococcaceae at the family level; *Odoribacter, unidentified_Ruminococcaceae, Catenisphaera, Bilophila*, and *unidentified Lachnospiraceae* at the genus level; *Porphyromonadaceace bacterium_DJF_B175* at the species level (Figures 6A,B). We further compared the F/B ratio between the two groups and found that the F/B ratio was significantly increased in the AOM/DSS + Abx + Akk group compared to the AOM/DSS + Abx group (Figure 6C).

It is intriguing that the AOM injection in the barren intestine after the antibiotic treatment dramatically boosted the growth of *A. muciniphila* as its relative abundance was increased by 160% in the AOM/DSS + Abx group and 786% in the AOM/DSS + Abx + Akk group on week 2 compared with their respective levels on week 1 (Figure 6D). However, the following DSS treatment sharply decreased the relative abundance of *A. muciniphila* in the AOM/DSS + Abx and AOM/DSS + Abx + Akk groups (Figure 6D). This observation was in parallel with what was observed on the AOM/DSS group (Figure 4F). Notably, the replenishment of viable *A. muciniphila* during the recovery phase after DSS drinking did not counteract the reducing effect of DSS administration on the relative abundance of *A. muciniphila* (Figure 6D).

Hence, the post-antibiotic *A. muciniphila* replenishment figured a distinguished microbial fingerprint during inflammation-associated tumorigenesis.

### *Akkermansia muciniphila* replenishment after antibiotic treatment influenced bile acids metabolism during tumorigenesis

**FIGURE 7:**
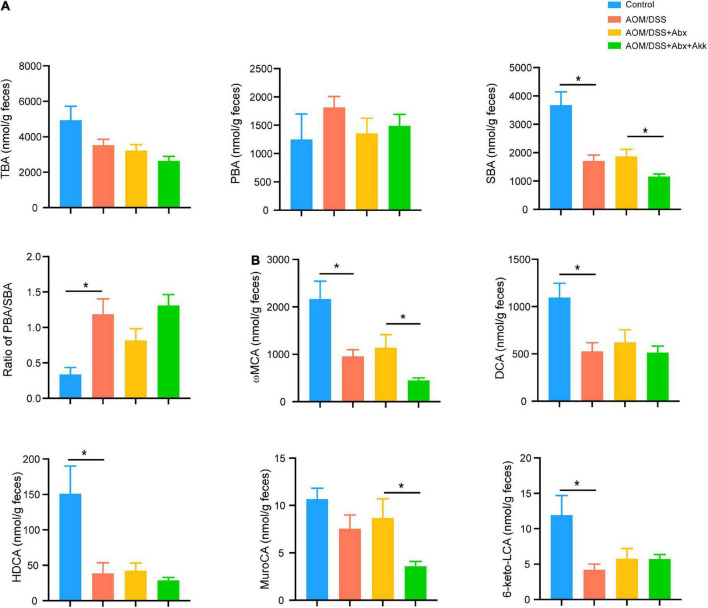
*Akkermansia muciniphila* administration impacted BA metabolism in the development of CAC. **(A)** Fecal levels of TBA, PBA, and SBA, and ratio of PBA/SBA in the four groups (week 12). **(B)** Levels of ωMCA, DCA, HDCA, muroCA, and 6-keto-LCA in the four groups. Data are shown as means ± SEM. *P < 0.05.

Targeted UPLC-MS/MS was performed to analyze the fecal BA. Compared to Control group, AOM/DSS group showed a significantly decreased level of secondary BA (SBA) by two times and an increased ratio of primary BA (PBA) to SBA by 3.5 times (Figure 7A). Although the level of total BA (TBA) showed a downtrend (4,923 ± 792.7 in Control vs. 3,523 ± 334.9 in AOM/DSS) and the level of PBA showed an uptrend (1,249 ± 450 in Control vs. 1,816 ± 191.4 in AOM/DSS), they showed no significant difference (Figure 7A). The levels of TBA, PBA, and SBA and the ratio of PBA/SBA were similar between the AOM/DSS and AOM/DSS + Abx groups (Figure 7A). Nevertheless, the post-antibiotic administration of *A. muciniphila* in AOM/DSS + Abx + Akk group further reduced the concentration of SBA by 1.6 times in comparison with the AOM/DSS + Abx group (Figure 7A). Besides, the ratio of PBA/SBA was 1.6 times higher in the AOM/DSS + Abx + Akk group than in the AOM/DSS + Abx group although without a significant difference (Figure 7A).

Further analysis of the BA composition showed that in comparison with the Control group, the tumorigenesis in the AOM/DSS group decreased the levels of some SBAs like ω–muricholic acid (ωMCA), hyodeoxycholic acid (HDCA), deoxycholic acid (DCA), and 6-keto-lithocholic acid (6-keto-LCA) (Figure 7B). The AOM/DSS and AOM/DSS + Abx groups shared a similar BA profile (Figure 7B). Intriguingly, the post-antibiotic *A. muciniphila* supplementation in the AOM/DSS + Abx + Akk group decreased the concentrations of ωMCA and muroCA compared to the AOM/DSS + Abx group (Figure 7B).

### Post-antibiotic *Akkermansia muciniphila* administration impacted the metabolism of short chain fatty acids in the development of colitis-associated colorectal cancer

**FIGURE 8:**
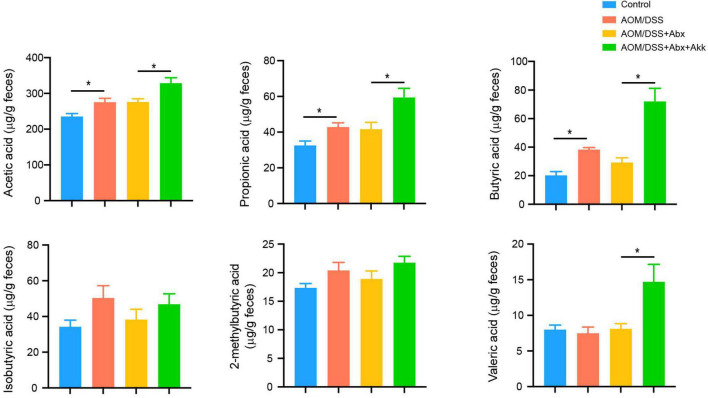
*Akkermansia muciniphila* administration impacted the metabolism of SCFAs in the development of CAC. Fecal SCFA levels (acetic acid, propionic acid, butyric acid, isobutyric acid, 2-methylbutyric acid, and valeric acid) in the four groups (week 12). Data are shown as means ± SEM. *P < 0.05.

In addition, we further conducted targeted GC-MS to analyze fecal SCFAs. Compared to the Control group, the inflammation-associated tumorigenesis in the AOM/DSS group increased the concentrations of acetic acid, propionic acid, and butyric acid (Figure 8). A similar profile of the six tested SCFAs was observed between the AOM/DSS + Abx and AOM/DSS groups (Figure 8). The levels of acetic acid, propionic acid, butyric acid, and valeric acid were increased in the AOM/DSS + Abx + Akk group compared to the AOM/DSS + Abx group, but the two groups shared similar concentrations of 2-methylbutyric acid and isobutyric acid (Figure 8).

In summary, the post-antibiotic supplementation with *A. muciniphila* during the tumorigenesis further affected the metabolism of BAs and SCFAs apart from the gut microbiota.

### Correlations among tumor formation indicators, intestinal barrier indexes, inflammatory cytokines, microbes, and metabolites

**FIGURE 9:**
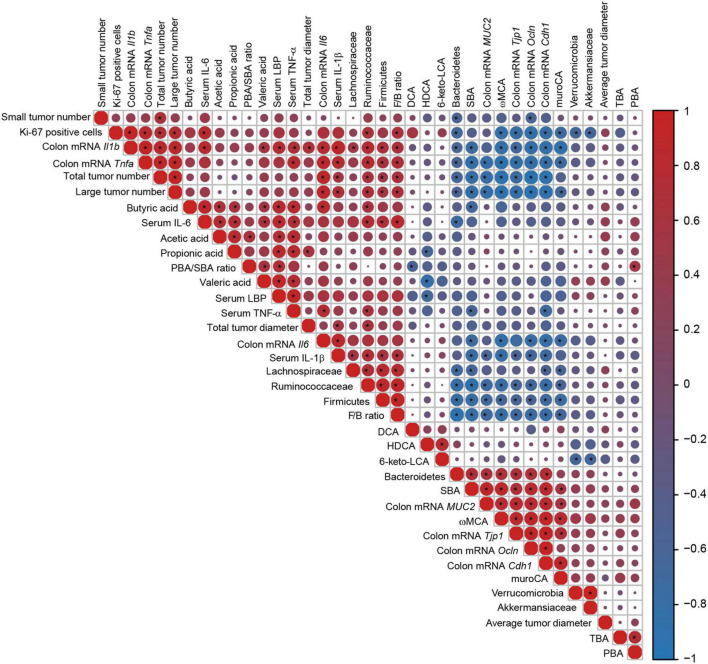
Correlations heatmap of the tumor formation indicators, intestinal barrier indexes, inflammatory cytokines, microbes, and metabolites. Color scale and circle size indicate the correlation degree, red indicates a positive correlation, and blue indicates a negative correlation. Asterisk indicates significance. *P < 0.05.

Correlations among tumor formation indicators, intestinal barrier indexes, inflammatory cytokines, microbes, and metabolites were analyzed by Spearman correlation analysis (Figure 9). Tumor burden and colonic cell proliferation were closely associated with inflammation and intestinal barrier damage. Large tumor numbers positively correlated with colonic mRNA levels of *Il1b, Il6*, and *Tnfa* and serum level of IL-1β, and were negatively associated with expressions of colonic *MUC2, Tjp1, Ocln*, and *Cdh1*. Percentage of Ki-67 positive cells had positive correlations with colonic transcriptional levels of *Il1b* and *Tnfa* and serum level of IL-6 but negative correlations with colonic mRNA levels of *Tjp1, Ocln*, and *Cdh1*. Furthermore, colonic levels of *Il1b, Il6*, and *Tnfa* and serum level of IL-1β negatively correlated with mRNA levels of *MUC2, Tjp1, Ocln*, and *Cdh1*. These indicated that inflammation (especially colonic inflammation) and impaired intestinal barrier had close correlation with tumorigenesis and tumor cell proliferation.

The relative abundance of Firmicutes and the F/B ratio showed positive correlations with large tumor numbers, Ki-67-positive cells, colonic mRNA levels of *Il1b* and *Tnfa*, and serum levels of IL-1β and IL-6. However, the relative abundance of Bacteroidetes had negative correlations with large tumor numbers, Ki-67-positive cells, and colonic mRNA levels of *Il1b* and *Tnfa*. The level of SBA had negative correlations with large tumor numbers, colonic mRNA levels of *Il1b, Il6*, and *Tnfa*, and serum levels of IL-1β and TNF-α but positive correlations with colonic transcriptional levels of *MUC2, Tjp1, Ocln*, and *Cdh1*. The level of ωMCA negatively correlated with large tumor numbers, percentage of Ki-67-positive cells, colonic mRNA levels of *Il1b, Tnfa*, and *IL6*, and serum level of IL-1β but positively correlated with colonic mRNA levels of *MUC2, Tjp1, Ocln*, and *Cdh1*.

## Discussion

In this study, we focused on the impact of *A. muciniphila*-involved post-antibiotic reconstitution of the microbial community on the development of CAC. A mouse model of CAC that mirrored what is seen in humans was developed in the context of antibiotic cocktail-induced disruption of intestinal microbiota, and *A. muciniphila* was administrated to mice during the development of CAC. The results showed that the *A. muciniphila* replenishment after the antibiotic pretreatment aggravated tumor growth as evidenced by increased large tumor numbers as well as higher colonic cell proliferation.

To better understand the mechanism behind the aggravation of CAC resulting from post-antibiotic *A. muciniphila* administration, we evaluated the intestinal barrier function, inflammatory environment, gut microbiota, and metabolism of BA and SCFAs.

Gut barrier dysfunction is an important factor in promoting colorectal carcinogenesis (Genua et al., 2021). We found that the AOM/DSS group showed reduced expression levels of *Tjp1, Ocln, Cdh1*, and *MUC2* in the colon tissues compared to the Control group, indicating a disrupted intestinal barrier in the progression of CAC. The post-antibiotic replenishment with *A. muciniphila* exacerbated the intestinal barrier damage as evidenced by further decrease in mRNA levels of *Tjp1, Ocln, Cdh1*, and *MUC2* in the AOM/DSS + Abx + Akk group with respect to the AOM/DSS + Abx group. Consequently, the destabilized tight junction and weakened mucosal barrier aggravated the bacteria-derived LPS translocation as indicated by the higher level of LBP in the AOM/DSS + Abx + Akk group than in the AOM/DSS + Abx group. The LPS in the gut and endotoxemia contributed to chronic intestinal and systemic inflammatory responses and promotion of colorectal carcinogenesis (Candelli et al., 2021). The colonic transcriptional levels of *Il1b, Il6*, and *Tnfa* and serum levels of IL-1β, IL-6, and TNF-α in the AOM/DSS group were elevated during tumorigenesis. The post-antibiotic supplementation with *A. muciniphila* further increased the colonic and systemic levels of the inflammatory cytokines in the AOM/DSS + Abx + Akk group. Pro-inflammatory cytokines (IL-1β, IL-6, and TNF-α) took center roles in the initiation and progression of CRC (Liu et al., 2012; Ho et al., 2014). The activation of nuclear factor-κB (NF-κB) by TNF-α and IL-1β as well as the activation of signal transducer and activator of transcription (STAT)3 by IL-6 in a tumor microenvironment could potentiate colonic inflammation, induce uncontrolled cell proliferation, and further promote CRC progression (Waldner et al., 2012; De Simone et al., 2015; Voronov and Apte, 2015; Lin et al., 2020). Besides, we also observed close correlations among tumor burden, tumor cell proliferation, intestinal barrier damage, and inflammatory cytokines. Thus, the supplementation of *A. muciniphila* on the basis of severe microbial dysbiosis caused impaired gut barrier integrity, which helped create a disease-favorable environment. The consequent overproduction of key cytokines (IL-1β, IL-6, and TNF-α) played an important role in the exacerbated tumor burden in the AOM/DSS + Abx + Akk group. A substantial number of studies have described that *A. muciniphila* promoted colitis and CRC in mouse models (Seregin et al., 2017; Khan et al., 2020; Wang et al., 2022). Enriched *A. muciniphila* eroded the mucus layer, impairing the intestinal barrier, which in turn posed greater susceptibility to colitis in mice, and induced excessive secretion of inflammatory cytokines (IL-1β, IL-6, and TNF-α) (Desai et al., 2016; Seregin et al., 2017; Khan et al., 2020). However, it is worth noting that some scholars have also highlighted that mucosal erosion-induced barrier damage and the pro-inflammatory effects of *A. muciniphila* are certain context-dependent (Seregin et al., 2017; Zhang et al., 2021).

The post-antibiotic supplementation of *A. muciniphila* interfered with the evolution of gut microbiota and reshaped a distinguished microbial composition during the tumorigenesis compared to the AOM/DSS and AOM/DSS + Abx groups. This microbial structure was characterized by significantly increased relative abundances of Firmicutes, Lachnospiraceae, and Ruminococcaceae and decreased relative abundance of Bacteroidetes, which is accompanied by increased F/B ratio. F/B ratio is widely accepted as an indicator of intestinal homeostasis or dysbiosis (Stojanov et al., 2020). On the contrary, previous studies have demonstrated decreased Firmicutes abundance and F/B ratio in patients with IBD and CRC or mice with DSS-induced colitis (Wang et al., 2012; Duboc et al., 2013; Bian et al., 2019, 2020; Liu et al., 2020). The significantly increased F/B ratio in our study indicated severe microbial dysbiosis caused by the post-antibiotic *A. muciniphila* supplementation. Furthermore, we observed that the higher F/B ratio was positively correlated with tumor burden, cell proliferation, and colonic and serum inflammatory cytokines, suggesting a strong association between microbial dysbiosis and colonic tumorigenesis.

The microbiota is involved extensively in host metabolism and has been described as a virtual metabolic organ (O’Hara and Shanahan, 2006). Thus, we further analyzed the metabolism of SCFAs and BA. The results showed that the tumorigenesis in AOM/DSS group was accompanied by increased concentrations of acetic acid, propionic acid, and butyric acid compared to the Control group. Furthermore, the post-antibiotic supplementation of *A. muciniphila* in the AOM/DSS + Abx + Akk group further increased the concentrations of the metabolites in comparison to the AOM/DSS + Abx group. *A. muciniphila* could metabolize mucin into propionate and acetate, and the enriched Ruminococcaceae and Lachnospiraceae families in the AOM/DSS + Abx + Akk group are butyrate producers (Louis and Flint, 2009, 2017; de Vos, 2017). The SCFAs could cooperate with microbes and intestinal cells to promote epithelial barrier function (de Vos, 2017; Parada Venegas et al., 2019). The bacterial metabolites could suppress cancer cell proliferation and induce apoptosis of colon cancer cell lines *in vitro* (Velayos et al., 2010; Matthews et al., 2012). Researchers also reported that probiotics and synbiotics may exert their beneficial effects on colitis or CAC by increasing the production of SCFAs (Bian et al., 2019; Zhai et al., 2019; Cruz et al., 2020). Unfortunately, our study showed that the increased SCFAs neither counteract or reverse the greater tumor burden in the AOM/DSS + Abx + Akk group nor help improve the barrier damage. Nevertheless, it is worth noting that some studies also demonstrated the dose-dependent phenomenon of butyric acid in colonic cells that high concentration of butyrate could promote colonic tumorigenesis (Yoshida et al., 2011; Zhang et al., 2018), suggesting a controversial role of butyrate in colonic inflammation or tumorigenesis.

Bile acids, especially SBAs, have been observed to promote tumorigenesis in the progression of CRC (Bernstein et al., 2005, 2011; Jia et al., 2018). Long-term repeat exposure of the intestine to high physiological SBA concentration could lead to occurrence of CRC (Berr et al., 1989; Bayerdörffer et al., 1995; Pereira et al., 2004; Bernstein et al., 2005, 2011; Hara, 2015; Cao et al., 2017). DCA is one of the SBAs, and its high level in the intestinal lumen has a potential pro-carcinogenic effect (Bernstein et al., 2011; Saracut et al., 2015; Cao et al., 2017). However, our study showed a discordant result that the concentrations of SBAs and DCA were decreased in tumor-bearing mice. The post-antibiotic administration of *A. muciniphila* further decreased the level of SBAs, and lower level of SBAs were negatively correlated with large tumor numbers and colonic and systemic pro-inflammatory cytokines. A study found that patients with active IBD exhibited low levels of stool SBAs and suggested that it was the loss of anti-inflammatory effects after the reduction of SBA that exacerbated the colonic inflammatory progression (Duboc et al., 2013). Another study also showed that treatment with DCA promoted an anti-inflammatory profile in murine colitis models (Sinha et al., 2020). These seem to contradict with the cancer-promoting properties of SBAs and DCA. However, what Jia et al. (2018) analyzed in their review may help explain our results to some degree. They proposed that high-fat diet (HFD) intervention would first increase the levels of SBA, but that the following bacterial dysbiosis would lead to decrease in SBA levels and subsequent Farnesoid X receptor (FXR) dysfunction and finally induce high proliferation of colon cells, contributing to colon tumorigenesis. However, it is more related to obesity-related CRC. Currently, most studies on the impact of BAs on the development of CRC have not been classified by a specific etiology of carcinogenesis, which may lead to the contradictory conclusions of the studies. ωMCA is a murine-specific SBA formed by enzymatic conversion of βMCA by a variety of intestinal microorganisms like Clostridium. Our study found a decrease in the fecal level of ωMCA in the AOM/DSS group compared to the Control group, and the *A. muciniphila* administration after the antibiotic pretreatment further reduced its level in the AOM/DSS + Abx + Akk group compared to the AOM/DSS + Abx group. The correlation analysis indicated that ωMCA level was negatively correlated with the tumor burden and colonic cell proliferation of CAC.

Previously, Suez et al. (2018) have found that probiotic administration after antibiotic treatment hindered the reconstitution of the gut microbiome and even led to poorer microbial composition. What we found is consistent with their study and confirmed that probiotics are not always effective. The disturbed natural evolution of the microbial community and subsequent changes in microbial metabolism would pose a great threat to host health and even aggravate disease progression. Thus, maintaining the equilibrium of the whole microecosystem is more important to host health than replenishing single beneficial microbes.

There are still some limitations in our study that need to be further addressed. Viable *A. muciniphila* was used for administration in our research. Plovier et al. (2017) reported that pasteurized *A. muciniphila* recapitulated and even enhanced the effect of *A. municiphila*, Therefore, investigating the effect of pasteurized *A. muciniphila* on the Abx-CAC model would help further understand the mechanism. Since the main purpose of this study was the impact of *A. muciniphila*-participated post-antibiotic gut microbial reconstruction on the development of CAC, we did not treat the CAC mice with *A. muciniphila*. So far, the characteristics of *A. muciniphila* in CRC are complicated. Some studies showed a correlation of *A. muciniphila* with increased tumor burden, and even the harmful effects of this bacterium on the progression of colonic tumorigenesis (Weir et al., 2013; Baxter et al., 2014; Wang et al., 2022), while some studies reported a protective potential of *A. muciniphila* (Wang et al., 2020; Fan et al., 2021). Thus, the direct contribution of *A. muciniphila* to CRC needs to be deeply investigated. In addition, more research is needed to further dissect the specific role of SCFAs and BA in CRC.

## Conclusion

Our study showed that post-antibiotic *A. muciniphila* replenishment increased the colonic tumor burden in mice, and its malign effects may be exert by aggravating intestinal barrier damage and colonic and systemic inflammation and interfering with reconstitution of the intestinal microbiota and its metabolic function. Further studies devoted to clarifying the specific underlying mechanism between *A. muciniphila* and CRC would be appreciated. Importantly, we need to keep in mind that probiotics should be used with caution, particularly after antibiotic treatment.

## Data availability statement

The datasets generated for this study can be found in the NCBI at the following link: https://www.ncbi.nlm.nih.gov/sra/?term=PRJNA748579.

## Ethics statement

The animal study was reviewed and approved by the Animal Care and Use Committee of The First Affiliated Hospital, School of Medicine, Zhejiang University.

## Author contributions

KW, WW, QW, and LLi designed the experiments. KW, LY, and XB conducted the experiments. KW, RY, and JX analyzed the data. SH helped KW with the figures. KW wrote the manuscript. LLv and XJ helped to revise the manuscript. All authors reviewed, contributed to the manuscript, and approved the submitted version.
