# Supplementary material for: The negative effect of Akkermansia muciniphila-mediated post-antibiotic reconstitution of the gut microbiota on the development of colitis-associated colorectal cancer in mice
Source: Front Microbiol. 2022 Oct 14;13:932047. doi: 10.3389/fmicb.2022.932047 (PMC9614165; doi:10.3389/fmicb.2022.932047)
Supplement: Supplementary file 1 [file Data_Sheet_1.docx]

Supplementary Table 1 Primers applied for RT-PCR

| Gene | Forward Sequence (5’-3’) | Reverse Sequence (5’-3’) |
| --- | --- | --- |
| *Gapdh* | TGCGACTTCAACAGCAACTC | ATGTAGGCAATGAGGTCCAC |
| *Tjp1* | GCCGCTAAGAGCACAGCAA | GCCCTCCTTTTAACACATCAGA |
| *Ocln* | GGAGGACTGGGTCAGGGAAT | CGTCGTCTAGTTCTGCCTGT |
| *Cdh1* | CAGGTCTCCTCATGGCTTTGC | CTTCCGAAAAGAAGGCTGTCC |
| *MUC2* | ATGCCCACCTCCTCAAAGAC | GTAGTTTCCGTTGGAACAGTGAA |
| *Tnfa* | CCCTCACACTCAGATCATCTTCT | GCTACGACGTGGGCTACAG |
| *Il6* | TAGTCCTTCCTACCCCAATTTCC | TTGGTCCTTAGCCACTCCTTC |
| *Il1b* | GAAATGCCACCTTTTGACAGTG | CTGGATGCTCTCATCAGGACA |

Supplementary Table 2 Comparison of the gut microbial composition of Control, AOM/DSS, AOM/DSS+Abx, AOM/DSS+Abx+Akk groups at week 1

|  | Adonis | |
| --- | --- | --- |
| Week 1 | R2 | Pr(>F) |
| Control *vs.* AOM/DSS | 0.19121 | 0.01 |
| Control *vs.* AOM/DSS+Abx | 0.49922 | 0.002 |
| Control *vs.* AOM/DSS+Abx+Akk | 0.6145 | 0.001 |
| AOM/DSS *vs.* AOM/DSS+Abx | 0.5218 | 0.001 |
| AOM/DSS *vs.* AOM/DSS+Abx+Akk | 0.66544 | 0.001 |
| AOM/DSS+Abx *vs.* AOM/DSS+Abx+Akk | 0.20089 | 0.014 |

Week 1: after seven days of antibiotics or drinking water treatment.

|  | Cytokine conc (pg/ml) in group | | | |
| --- | --- | --- | --- | --- |
| Inflammatory cytokine | Control | AOM/DSS | AOM/DSS+Abx | AOM/DSS+Abx+Akk |
| Eotaxin | 155.135±5.2701 | 205.4663±10.0681^*^ | 158.1817±7.2889^$^ | 162.1789±9.913 |
| G-CSF | 408.5429±62.5217 | 715.5257±61.7848^*^ | 607.9825±146.7343 | 624.3617±44.9533 |
| IFN-γ | 11.3163±1.7176 | 13.7943±2.1176 | 10.8386±1.3328 | 13.015±1.434 |
| IL-1α | 3.04±1.5698 | 8.25±1.6383 | 9.475±0.9421 | 12.6957±1.3072 |
| IL-1β | 1.5557±0.1093 | 2.0344±0.167^*^ | 2.115±0.0869 | 2.7644±0.1809^#^ |
| IL-2 | 2.1133±0.4467 | 2.338±0.2963 | 2.0025±0.3799 | 3.2117±0.4311 |
| IL-3 | 13.0771±0.6191 | 13.6043±0.8334 | 11.8625±0.7287 | 15.0914±0.929^#^ |
| IL-4 | 0.5175±0.0425 | 1.506±0.1393^*^ | 1.2125±0.1751 | 1.426±0.2612 |
| IL-5 | 1.5±0.3247 | 2.7167±0.3215^*^ | 2.5425±0.4057 | 3.2438±0.4524 |
| IL-6 | 5.05±0.5679 | 8.9313±0.8368^*^ | 8±1.3798 | 15.206±1.031^#^ |
| IL-9 | 14.4967±1.6986 | 13.4575±4.1872 | 7.91±1.6088 | 17.8267±1.1071^#^ |
| IL-10 | 16.776±4.032 | 13.104±1.0684 | 10.0733±2.193 | 15.5967±2.1573 |
| IL-12(p40) | 977.1414±70.3365 | 1242.8656±74.9845^*^ | 963.5238±98.3784^$^ | 1399.3833±99.1614^#^ |
| IL-12(p70) | 64.5529±14.727 | 74.7057±11.7339 | 52.7171±10.3232 | 64.6333±9.6028 |
| IL-13 | 38.73±8.8999 | 205.1813±135.9184 | 63.8238±5.5907 | 64.7178±11.4004 |
| IL-17A | 62.4188±4.9635 | 60.0775±7.2681 | 42.9138±3.1219 | 77.5189±6.9016^#^ |
| KC | 45.74±1.7457 | 64.5675±7.2794 | 52.6013±6.0611 | 113.3267±36.4996^#^ |
| MCP-1 | 80.1325±7.7884 | 116.3775±12.0841 | 92.1663±9.5456 | 341.5911±144.0567^#^ |
| MIP-1a | 1.705±0.1119 | 1.88±0.1163 | 3.6375±0.406^$^ | 3.6911±0.5993 |
| MIP-1b | 66.105±11.5704 | 97.2013±21.3542 | 57.0775±14.0709 | 137.7211±31.857 |
| RANTES | 178.4814±20.6177 | 191.6111±16.1056 | 185.3513±16.1848 | 164.1433±10.3507 |
| TNF-α | 25.925±0.7464 | 31±1.3799^*^ | 31.478±0.478 | 38.5238±2.0505^#^ |

Supplementary Table 3 Serum inflammatory cytokine levels in Control, AOM/DSS, AOM/DSS+Abx, AOM/DSS+Abx+Akk groups

Data are shown as the mean ± SEM, ^*^, P<0.05 compared to the Control group; ^$^, P<0.05, compared to the AOM/DSS group; ^#^, P<0.05, compared to the AOM/DSS+Abx group.

Supplementary Table 4 Comparison of gut microbial composition in different phases of tumorigenesis in the AOM/DSS group

|  | Adonis | |
| --- | --- | --- |
|  | R2 | Pr(>F) |
| Week 2 *vs.* Week 1 | 0.43289 | 0.001 |
| Week 5 *vs*. Week 2 | 0.49388 | 0.001 |
| Week 8 *vs*. Week 5 | 0.37362 | 0.001 |
| Week 12 *vs*. Week 8 | 0.57622 | 0.001 |

Week 1: the baseline; week 2: after AOM injection; week 5: after the first cycle of DSS treatment; week 8: after the second cycle of DSS treatment; week 12: after the third cycle of DSS treatment and before sacrifice.

Supplementary Table 5 Comparison of the gut microbial compositions of Control, AOM/DSS, AOM/DSS+Abx, AOM/DSS+Abx+Akk groups during the process of tumorigenesis

|  | Adonis | |
| --- | --- | --- |
|  | R2 | Pr(>F) |
| **Week 2** |  |  |
| Control *vs.* AOM/DSS | 0.25473 | 0.002 |
| Control *vs.* AOM/DSS+Abx | 0.50402 | 0.001 |
| Control *vs.* AOM/DSS+Abx+Akk | 0.64581 | 0.001 |
| AOM/DSS *vs.* AOM/DSS+Abx | 0.30662 | 0.001 |
| AOM/DSS *vs.* AOM/DSS+Abx+Akk | 0.45822 | 0.001 |
| AOM/DSS+Abx *vs.* AOM/DSS+Abx+Akk | 0.22644 | 0.001 |
| **Week 5** |  |  |
| Control *vs.* AOM/DSS | 0.28885 | 0.002 |
| Control *vs.* AOM/DSS+Abx | 0.41117 | 0.001 |
| Control *vs.* AOM/DSS+Abx+Akk | 0.54605 | 0.001 |
| AOM/DSS *vs.* AOM/DSS+Abx | 0.41094 | 0.001 |
| AOM/DSS *vs.* AOM/DSS+Abx+Akk | 0.61602 | 0.001 |
| AOM/DSS+Abx *vs.* AOM/DSS+Abx+Akk | 0.71866 | 0.001 |
| **Week 8** |  |  |
| Control *vs.* AOM/DSS | 0.51571 | 0.001 |
| Control *vs.* AOM/DSS+Abx | 0.4104 | 0.001 |
| Control *vs.* AOM/DSS+Abx+Akk | 0.52416 | 0.001 |
| AOM/DSS *vs.* AOM/DSS+Abx | 0.11794 | 0.002 |
| AOM/DSS *vs.* AOM/DSS+Abx+Akk | 0.26678 | 0.001 |
| AOM/DSS+Abx *vs.* AOM/DSS+Abx+Akk | 0.283 | 0.001 |
| **Week 12** |  |  |
| Control *vs.* AOM/DSS | 0.18102 | 0.005 |
| Control *vs.* AOM/DSS+Abx | 0.20218 | 0.002 |
| Control *vs.* AOM/DSS+Abx+Akk | 0.22883 | 0.001 |
| AOM/DSS *vs.* AOM/DSS+Abx | 0.05419 | 0.332 |
| AOM/DSS *vs.* AOM/DSS+Abx+Akk | 0.24478 | 0.001 |
| AOM/DSS+Abx *vs.* AOM/DSS+Abx+Akk | 0.23316 | 0.001 |

Week 2: after AOM injection; week 5: after the first cycle of DSS treatment; week 8: after the second cycle of DSS treatment; week 12: after the third cycle of DSS treatment and before sacrifice.
